# False positive finding on whole-body iodine-131 scan secondary to contaminated face mask: an uncommon peril in current COVID pandemic

**DOI:** 10.22038/AOJNMB.2022.63815.1449

**Published:** 2023

**Authors:** Anupriya Anwariya, Piyush Aggarwal, Ashwani Sood, Nitheesh Tigapuram, Sarika Prashar

**Affiliations:** Department of Nuclear Medicine, Postgraduate Institute of Medical Education and Research, Chandigarh, India

**Keywords:** COVID-19, Iodine-131, Whole Body Imaging, Masks, Thyroid cancer

## Abstract

Covid-19 has changed the practice of present-day medicine. Social-distancing, hand-sanitation and use of face-mask are important measures taken against its spread. Post-thyroidectomy whole-body diagnostic I-131 scan is an important preliminary investigation for risk stratification and further management in thyroid cancer. False positive findings on diagnostic scan are not uncommon and must be evaluated to avoid unnecessary work-up and treatment. Clinical and biochemical correlation with adjunct SPCET/CT imaging may differentiate true from false-positive lesions. We report a case of unusual false positive linear neck tracer on whole-body diagnostic I-131 scan due to the use of an I-131 contaminated face mask.

## Introduction

 The era of COVID-19 has brought with it many new challenges in the everyday practice of medicine affecting almost all specialties and subspecialties. False-positive uptake on radioactive iodine scans is not uncommon, and such findings should be interpreted with caution. We report one such case of a 51-year-old man with incidental neck uptake on a diagnostic iodine-131 (I-131) scan, which on careful evaluation turned out to be due to his face mask being contaminated with sweat and saliva. Although following COVID appropriate behavior is crucial to prevent its spread, care must be taken to replace the face mask during scan acquisition to avoid such false-positive findings.

## Case Report

**Figure 1:**
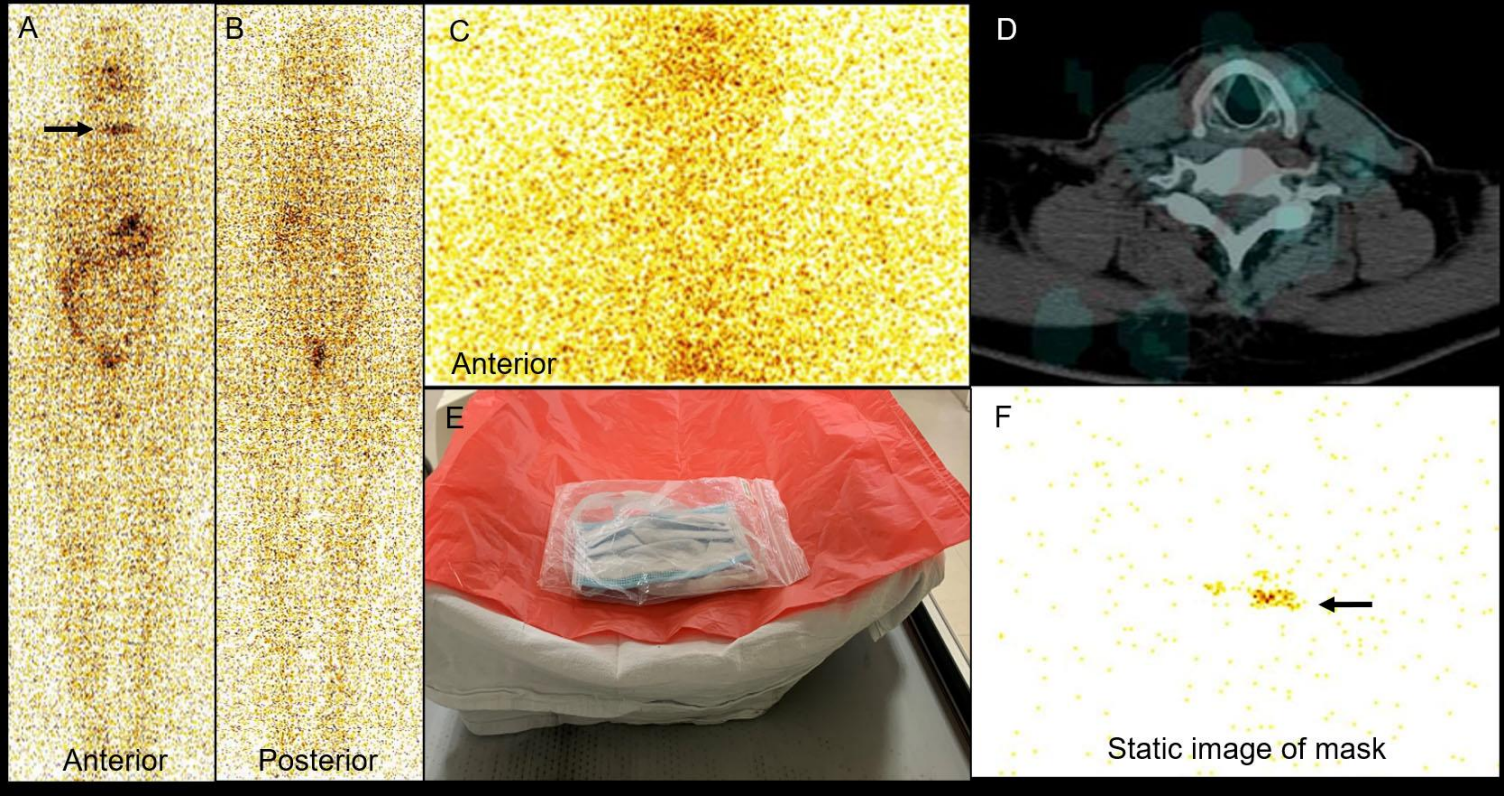
Anterior (**A**) and posterior (**B**) views of the planar whole-body diagnostic Iodine-131 scan, showing linear focus of tracer activity in the neck region on anterior view (**A, arrow**). Static anterior planar image of the head region (**C**) after cleaning the area with corresponding transaxial hybrid SPECT/CT image (**D**) showing no abnormal focus of tracer activity. The mask was wrapped in a plastic bag to avoid contamination (**E**) and its static planar image revealed a linear focus of tracer activity (**F, arrow**)

 A 51-year-old man with papillary carcinoma of thyroid, post-total thyroidectomy with bilateral selective lymph node dissection with pathological stage of pT2N1Mx was referred to our department for further management. His post-operative serum thyroid stimulating hormone (TSH), thyroglobulin and anti-thyroglobulin antibody levels of patient were 84.9 µIU/ml (normal: 0.4-4.0 µIU/ml), 0.45 ng/ml (normal: 1.40–29.2 ng/ml), and 1092 IU/ml (normal < 20 IU/ml) respectively, during the peak of COVID-19. He underwent a whole-body radioiodine (I-131) diagnostic scan and images acquired after 48 hours of radioiodine (~1.5 mCi) administration (Figure 1) revealed abnormal linear tracer activity in the anterior neck region (Figure 1A, arrow). The unusual presentation of linear tracer distribution at the surgical site led to the suspicion of contamination. The patient was then asked to remove the mask and clean the neck region with water, and the subsequent anterior planar image of the head and neck region along with single photon emission computed tomography/ computed tomography (SPECT/CT) showed no abnormal tracer activity in the neck region. Careful scrutiny revealed that the patient had been wearing the same surgical face mask, probably soaked in sweat/saliva, during the image acquisition, which he had worn at the time of I-131 dose administration. The patient had a long beard and gave history of lowering and resting the face mask over the neck region during the scan acquisition, probably resulting in linear tracer uptake in the lower neck region because of contamination. To confirm this suspicion, the contaminated surgical face mask was imaged under the gamma camera using adequate precautions by wrapping the mask in a plastic bag to avoid contamination which showed a linear streak of faint tracer activity (Figure 1F, arrow). The false-positive scan finding was possibly secondary to the collection of I-131 contaminated sweat/salvia over a period of two days in the lower margin of the surgical face mask.

## Discussion

 False-positive findings on whole-body radioiodine scans are not uncommon. Use of a face mask contaminated with secretions of sweat/saliva may lead to false-positive tracer distribution and misinterpretation of diagnostic and post-therapy whole-body radioiodine scans (1). Prompt suspicion of contamination because of an uncommon pattern of tracer distribution along with the detailed history from the patient is crucial in the correct interpretation of scans. Sodium iodide symporters (NIS), play a major role in the uptake of iodine through the basolateral membrane of thyroid follicular cells, which is required for the biosynthesis of thyroid hormones. NIS is also expressed in the epithelium of other non-thyroidal cells, where it is not regulated by TSH, like salivary glands, 

lacrimal glands, sweat glands, lactating mammary glands, and gastric mucosa. The presence of radioiodine in body fluids and therefore contamination by physiological secretions is quite common, which can lead to false-positive uptake on I-131 whole body scan (2-7). Hybrid imaging using SPECT/CT has incremental value in such cases with discordant biochemical and imaging findings [8]. In the era of COVID-19, this false positive finding secondary to contaminated face masks has resulted in the practice of replacing the old mask with a new mask before the acquisition of radioiodine scans in our department.

## Conflict of Interest Statement

 The authors have no conflicts of interest to declare.

## Funding Sources

 The authors did not receive any funding for the present work.

## Patient consent statement

 The patient/ the next of kin have given written informed consent to publish the case including the publication of images.
